# Natural Agents Targeting Mitochondria in Cancer

**DOI:** 10.3390/ijms21196992

**Published:** 2020-09-23

**Authors:** Shalini Mani, Geeta Swargiary, Keshav K. Singh

**Affiliations:** 1 Centre for Emerging Diseases, Department of Biotechnology, Jaypee Institute of Information Technology, A-10, Sector 62, Noida 201307, UP, India; shalini.mani@jiit.ac.in (S.M.); swargiarygeeta@gmail.com (G.S.); 2 Department of Genetics, University of Alabama at Birmingham, Birmingham, AL 35294, USA

**Keywords:** cancer, mitochondria, mitocans, anticancer herbs, natural agents

## Abstract

Mitochondria are the key energy provider to highly proliferating cancer cells, and are subsequently considered one of the critical targets in cancer therapeutics. Several compounds have been studied for their mitochondria-targeting ability in cancer cells. These studies’ outcomes have led to the invention of “mitocans”, a category of drug known to precisely target the cancer cells’ mitochondria. Based upon their mode of action, mitocans have been divided into eight classes. To date, different synthetic compounds have been suggested to be potential mitocans, but unfortunately, they are observed to exert adverse effects. Many studies have been published justifying the medicinal significance of large numbers of natural agents for their mitochondria-targeting ability and anticancer activities with minimal or no side effects. However, these natural agents have never been critically analyzed for their mitochondria-targeting activity. This review aims to evaluate the various natural agents affecting mitochondria and categorize them in different classes. Henceforth, our study may further support the potential mitocan behavior of various natural agents and highlight their significance in formulating novel potential anticancer therapeutics.

## 1. Introduction

As per World Health Organization (WHO) records, cancer is one of the world’s most deadly diseases. WHO estimated that 9.6 million people worldwide died due to cancer in 2018 [1]. Despite several research and therapeutics efforts, the exact cure for cancer is still elusive, and cancer has successfully invaded millions of lives. Different cancer treatment options like surgery, immunotherapy, hormone therapy, chemotherapy, and radiotherapy are available. Still, these treatments could only control the disease for a short duration, later worsening the patient’s condition. Researchers have aimed to exploit cancer cell vulnerabilities using various approaches, including identifying gene targets, discovering novel compounds, and developing technologies such as nanoparticles for selective targeting which can be effective against cancer growth [2,3,4]. Unfortunately, cancer cells develop resistance to chemotherapy by resisting apoptosis and/or exporting the drugs outside the cell.

To proliferate in hostile conditions, cancer cells undergo metabolic reprogramming to meet their energetic needs. This change has been identified as an important hallmark of cancer and a potential vulnerability targeted to fight against cancer [5]. Otto Warburg, in 1930, described the metabolic shifting that occurs in the mitochondria of cancer cells and called it the Warburg effect. The Warburg effect showed that cancer cells, unlike normal cells, rely heavily on oxidative glycolysis for their energy requirements [6]. Oxidative glycolysis in cancer cells serves as a source of glutamine, and glucose supplies most of the carbon, nitrogen, free energy, and reducing equivalents necessary to support cell growth and division. Thus, oxidative glycolysis confers advantages to cancer cells by allowing a fast conversion of nutrients into biomass to enable cell proliferation [7,8,9].

Cancer cells have higher metabolic needs and antioxidant defenses compared with healthy cells [10]. Along with oxidative glycolysis, mitochondria are also crucial for the proliferation of cancer cells. [11]. Additionally, the heavy dependence of some cancers on oxidative phosphorylation (OXPHOS) for their ATP needs further highlights the importance of mitochondria in cancer [12,13,14]. In both scenarios, mitochondria-targeting treatments can disrupt OXPHOS machinery and lead to cancer cell death. [14]. Recent reports have also indicated that mitochondria are vital contributors in tumorigenesis through the process of metabolic reprogramming, mitochondrial depolarization, oxidative signaling, generation of ROS, and production of metabolites that enhance oncogenesis [15,16,17,18,19].

To meet the energy demands, cancer cells massively rely on oxidative glycolysis, which also leads to the upregulation of glucose transporters [20,21]. The upregulated glucose transporters encourage the hyperproliferation of cancer cells by supplying glucose in excess. The high glycolytic rate generates a lot of metabolic acids such as lactate and pyruvate; however, cancer cells have a higher intracellular pH (≥7.4) and a lower extracellular pH (~6.7–7.1) [22]. These pH conditions in cancer cells are just a complete inversion of the pH gradient compared to normal cells [23,24,25]. This hallmark of cancer cells lies in the overactivation of plasma membrane ion pumps and transporters that extrude protons and intrude other ions. The most common transporters expressed in cancer cells are H^+^/Na^+^ exchanger; voltage-gated proton channel; H^+^/K^+^ ATPase; H^+^/monocarboxylate cotransporters and Na^+^/HCO^3−^ cotransporter [26]. The lactate ions are rapidly extruded outside the cancer cells by the H^+^/monocarboxylate cotransporters. Due to high intracellular pH, these cells are lenient for cell proliferation and escape apoptosis [25]. Such a reversal in pH gradient further helps the metastatic progression of cancer cells [24].

The mitochondria of cancer cells are hyperpolarized in comparison to the normal cells [21]. This hyperpolarization may be due to the increased intracellular Ca^2+^ levels and upregulation of anti-apoptotic Bcl-2 protein in the cancer cells [27,28]. The change in expression levels of different adenine nucleotide translocase (ANT) in cancer cells may also contribute to the hyperpolarization of mitochondria [29]. Therefore, including change in acidification of cytosol, the higher mitochondrial membrane potential is also observed as other mitochondria-associated hallmarks of cancer cells. Hyperpolarization of the mitochondrial membrane potential in cancer cells may enhance the selective transfer of different mitochondria-targeting drugs into the cancer cells, which may induce apoptosis of the cancer cells. Studies also suggest that apoptosis in the cells can be triggered by damaging the DNA, elevating the oxidative stress, and depolarizing the mitochondria [30]. Another characteristic of cancer cell metabolism is the relatively higher ROS levels observed in the tumor cells than healthy cells. Excessive ROS levels in the cells are toxic and cause cell death [31,32]. However, in cancer cells, this higher ROS level contributes to transformation, proliferation, survival, migration, invasion, and metastasis of the tumor cells [33]. Recently, researchers have focused on exploiting these vulnerabilities and differences in ROS level to develop novel therapeutic agents that may trigger apoptosis in the cancer cells and eliminate the disease.

Mitochondria contain several pro-apoptotic factors and can trigger the programmed cell death pathway [34]. An attempt to design better therapy has led to identifying compounds known as the “mitocans, “which are exclusively designed to target the cancer cells’ mitochondria. The formulation of mitocans has primarily focused on elevating oxidative stress and destabilization of the mitochondrial membrane in cancer cells [35,36,37], ultimately shutting down the cancer cells mitochondria-mediated apoptosis.

The different classes of mitocans are proposed to target the mitochondria in different ways [38], but they ultimately aim to induce mitochondria-mediated apoptosis of the cancer cells. The classified mitocans are in various phases of clinical trials. However, unlike any other synthetic drugs, there is a possibility that those mitocans could also hold their side effects. To minimize or eradicate their plausible side effects, simultaneously an exploratory study is going on. Thus far, these studies suggested the utilization of anticancer herbs as a source of potential mitocans, which may be termed as the “Herbal mitocans” [39]. The interest on anticancer herbs arises from the fact that ancient times always relied on the medicinal herbs, which demonstrated excellent effectiveness on many diseases such as malaria, diarrhea, tuberculosis, pneumonia, and asthma [40]. So, why not formulate herbal mitocans with minimal or no side effects? There are a handful of natural agents that are reported to influence the mitochondria in cancer cells. We have also come across different herbal extracts, tagged with the preliminary evidence of affecting these cells’ mitochondria and possibly can be a source of potential herbal mitocans. Though the individual studies indicate the importance of these natural agents, however, there is a lack of systematic compilation of all the research outcomes and justification for their suitable mitocan class, which can further emphasize the need for functional validation of all such compounds/ extracts. Our current review is an attempt to fill these gaps in the research area of mitocans.

## 2. Mitocans: The Alternative Cancer Therapy

### 2.1. The Inception

**Figure 1:**
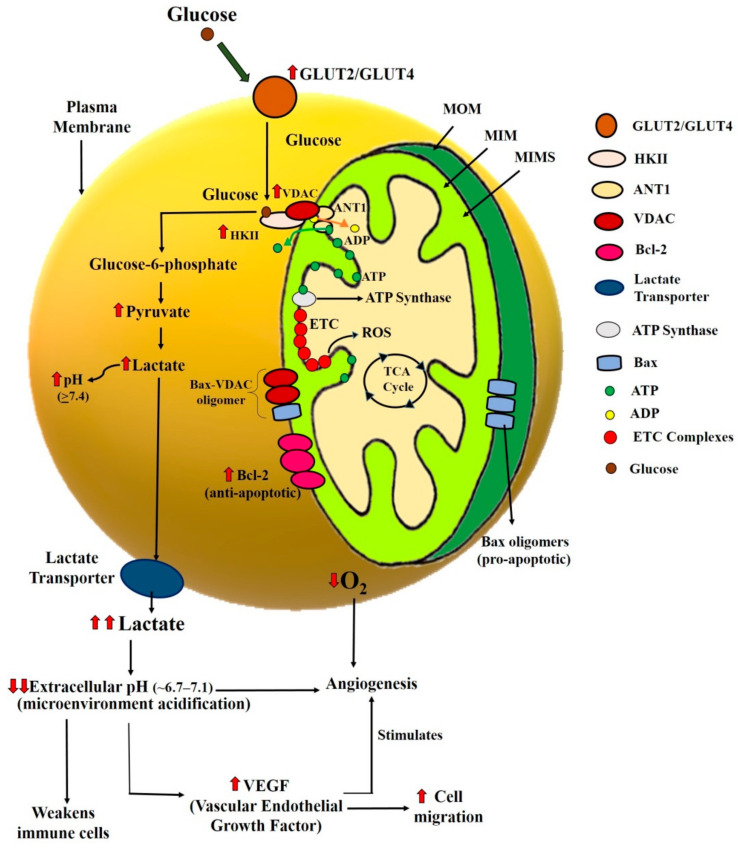
Illustration of the mechanism of cancer cell bioenergetics and the critical checkpoints in cancer cells. Glucose enters the cells via glucose transporters (GLUT2/GLUT4) and binds as a substrate to HK-II that utilizes ATP as another substrate for converting glucose to glucose six phosphate (G6P). G6P is further used for the glycolysis and OXPHOS for the production of ATP. This process of ATP production via glycolysis produces Lactate from its end product, pyruvate. In cancer cells, the constant activity of glycolysis leads to an increase in lactate that reduces the pH of the cancer cell environment. This whole phenomenon’s overall consequence is favorable for the cancer cell by stimulation of angiogenesis, increased VEGF, and an increase in cancer cell migration. Further, it also weakens the immune cells. MOM: mitochondrial outer membrane; MIM: mitochondrial inner membrane; MIMS: mitochondrial intermembrane space.

In cancer cells, the energy production process makes a way through the action of glycolysis to fulfill the energy demands [9]. Thus glycolytic rate is observed to be dramatically high in the early stage of cancer. As the process of glycolysis is linked and dependent on mitochondrial activity [41,42,43,44], mitochondria have been proposed to be one of the vital suspects in the etiology of cancer. Moreover, mitochondrial metabolism is also dependent on different types of proteins as well as biochemical processes. For instance, Bcl-2 family proteins regulate the mitochondrial permeability transition (MPT) pore opening, followed by the release of apoptogenic proteins from mitochondria to the cytosol and apoptosis [45,46,47]. Similarly, defects in the electron transport chain (ETC) and the tricarboxylic acid cycle (TCA cycle) that occurs within the mitochondria also affect the ATP and ROS levels. They may eventually affect cancer cell metabolism [48,49]. Therefore, defects in such proteins/processes could cause cellular stress in the cancer cell and bring them into mitochondria-mediated apoptosis [7]. By looking at the importance of mitochondria in cancer cells, different drug/compounds have been studied, either directly or indirectly targeting the mitochondria. In 2009, Ralph and Neuzil reviewed such compounds that we can target the cancer cells’ mitochondria in different ways and termed them as “mitocans” [50]. Cancer cells are metabolically and bioenergetically different from healthy cells; hence the broad aim of all the mitocans is to trigger either the cytostatic or cytotoxic effects on tumor cells by targeting the various checkpoints of cellular bioenergetics (Figure 1).

As per various studies, Hexokinase II (HKII), a key enzyme for the glycolytic pathway, is known to be overexpressed in cancer cells. Glucose transporters and voltage-dependent anion channel (VDAC) are also known to influence the glycolytic activity and overexpressed in cancer cells [51,52,53]. Figure 1 elaborates on the significance of these proteins in the survival of cancer cells and the importance of targeting such proteins to enhance specificity in cancer therapeutics.

Glucose transporters are the proteins located in the plasma membrane, and it is the gateway to the glycolytic pathway. Therefore, Glucose transporters GLUT2 and/or GLUT4 (depending on the cell types) overexpress and lead to higher glucose uptake in proliferating cancer cells [54,55,56]. Proliferating cells show a metabolic profile different from resting cells. This is further characterized by an increased glycolysis rate and an increase in lactic fermentation at the expense of OXPHOS. Various studies have suggested that to facilitate the transportation of ADP and ATP across the mitochondrial membrane, two types of ANT channels, ANT1 and ANT2, are present in mitochondria’s inner membrane [29]. ANT1 helps to transport ADP into the matrix and ATP (produced by ATP synthase) in the intermembrane space. In cancer cells, ANT1 is downregulated and instead, ANT2 is overexpressed in these cells. ANT2 also transports the same molecules, but transportation is facilitated in the opposite direction. ANT2 is predicted to export ADP from the matrix to the cytosol in exchange for ATP. In this way, the ATP synthase switches in the reverse mode: it hydrolyzes ATP and pumps protons across the inner membrane, thereby creating a state of hyperpolarization. This drop in ATP levels can also facilitate cytosolic glycolysis and further lactate production. The hyperpolarized state is predicted to protect the cancer cells from apoptosis, too [29].

The hyperactivated H^+^/monocarboxylate cotransporters export the lactate out of these cells [57] and acidify the cancer cells’ cellular microenvironment. This further leads to the weakening of the immune cells, increases the expression of vascular endothelial growth factor (VEGF), and thus stimulates the angiogenesis and cell migrations [57]. Angiogenesis is the lifeline for the cancer cell because it would supply oxygen for their growth and proliferation.

The massive reliance of the cancer cells on aerobic glycolysis results in the overexpression of the enzyme hexokinase, which catalyzes the first step of glucose metabolism [51,53]. Although there are four isoforms of hexokinases, only HKII plays a crucial role in cancer cell survival and proliferation. HKII binds to the VDAC at the outer mitochondrial membrane and facilitates their interaction with ANT. This interaction couples the aerobic glycolysis with the OXPHOS and enables the HKII to exchange ADP for ATP from the inner mitochondrial membrane, further increasing the glycolysis rate [58,59]. VDAC is also known to play a crucial role in apoptosis by releasing cytochrome c from mitochondria. However, in cancer cells, most of the VDAC is primarily occupied with HKII proteins, which eventually block the release of cytochrome c through VDAC [47]. The anti-apoptotic proteins are also highly expressed which hinders the mitochondria-mediated apoptosis in cancer cells [60]. All these changes in the expression of essential proteins involved in glycolytic pathways may promote cancer cell survival and, consequently, proliferation. From this mechanism, it is clear that inhibition of glucose transporters, HKII, VDAC, ANT, and lactate transporters can trigger cytostatic status in cancer cells and, hence, behave as promising targets of cancer therapy.

The cytotoxic effects of mitocans can mostly be exhibited by triggering the mitochondria-mediated apoptosis (Figure 1) in cancer cells. The Bcl-2 family proteins regulate the phenomenon of mitochondria-mediated apoptosis. The pro-apoptotic members of the Bcl-2 family (i.e., Bax and Bak) are activated upon stress-inducing stimuli [61]. The activated pro-apoptotic Bcl-2 family proteins oligomerize in the mitochondrial outer membrane by forming a pore to escape the mitochondria’s apoptotic molecules. On the other side, the anti-apoptotic Bcl-2 family proteins (i.e., Bcl-2 and Bcl-XL) regulate this intrinsic cell death pathway by preventing Bax and Bak’s activation. The anti-apoptotic Bcl-2 family proteins are highly expressed in cancer cells [62] and increase the mitochondrial outer membrane potential to inhibit the release of cytochrome c from the mitochondria [62]. Thus, to trigger cell death in cancer cells, the aim should be to reduce the mitochondrial membrane potential and trigger cytochrome c from mitochondria to cytosol, followed by cell death in cancer cells.

**Figure 2:**
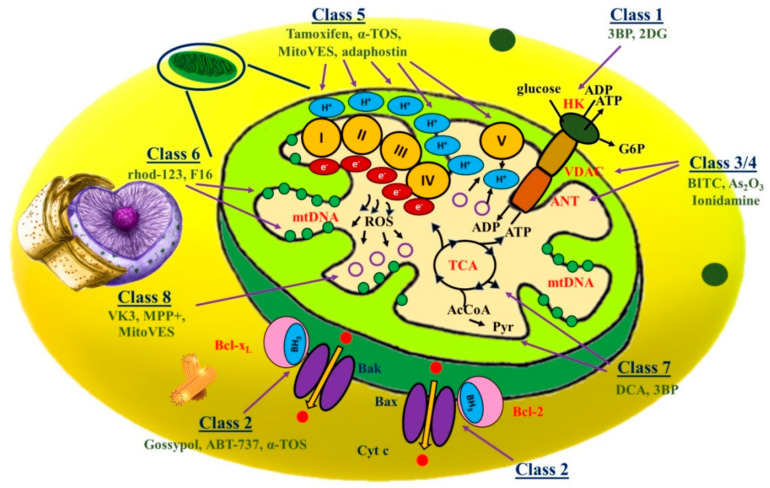
Different classes of mitocans and illustration of their molecular targets. Class 1: Hexokinase inhibitors; Class 2: BH3 mimetics and related agents that impair the function of the anti-apoptotic Bcl-2 family proteins; Class 3: Thiol redox inhibitors; Class 4: Agents targeting voltage-dependent anion channel (VDAC) and adenine nucleotide translocase (ANT); Class 5: Compounds targeting the mitochondrial electron transport chain; Class 6: Hydrophobic cations targeting the mitochondrial inner membrane (MIM); Class 7: Compounds that affect the tricarboxylic acid (TCA) cycle; and Class 8: Agents that interfere with mtDNA. Examples of each class are mentioned in the figure respective to their studies.

Different mitocans are proposed to exhibit either the cytostatic or cytotoxic effect; however, based upon their detailed action mode, mitocans were further divided under other groups [38,50] (Figure 2).

#### 2.1.1. Class 1 Mitocans: Hexokinase Inhibitors

As explained above, HKII is required at the very first step of glucose metabolism and are overexpressed in cancer cells; hence, targeting the HKII may uncouple the process of glycolysis and cause cell death. Recent research has focused on exploring those agents that can potentially inhibit the action of HKII, thereby inhibiting the function of glucose metabolism. For example, 2-deoxy-d-glucose (2-DG) and 3-bromopyruvate (3-BP) are the common synthetic mitocans that exhibit their cytotoxic effect on the cancer cells by inhibiting the HKII [63,64]. Benserazide is another mitocan that selectively inhibits HKII by specifically binding to HKII and inhibiting the enzymatic activity of HKII. Moreover, benserazide had reduced the glucose uptake, production of lactate, and intracellular ATP levels. As a result, the loss of membrane potential increases and causes apoptosis of the cancer cell [65].

As the class 1 mitocans primarily limit the glucose metabolism, the use of such compounds may be significantly important where the glucose metabolism is deregulated.

#### 2.1.2. Class 2 Mitocans: Compounds Targeting Bcl-2 Family Proteins

The anti-apoptotic Bcl-2 family proteins play a vital role in the inhibition of apoptosis of the cells. Therefore, they are a critical target in killing the cancer cells. This class of mitocans acts as the BH3 domain of the Bcl-2 family proteins. The anti-apoptotic Bcl-2 family proteins interact through the BH3 domain of the Bax and/or Bak and, as a result, prevent the BH3 domain from forming a pore in the mitochondrial outer membrane [66]. The pore in the mitochondrial outer membrane is formed by the oligomers of pro-apoptotic Bax or Bak proteins, so the increased expression of the anti-apoptotic proteins Bcl-2, Bcl-XL, or Mcl-1 interacting with the BH3 domain will protect the cancer cells from apoptosis.

Moreover, anti-apoptotic Bcl-2 family proteins are found to be overexpressed in cancer cells [67]. Therefore, agents that may act as or mimic the BH3 domain can target the interaction between the pro-apoptotic and anti-apoptotic Bcl-2 family proteins [68,69]. For example, gossypol, ABT-263, and α-tocopheryl succinate (α-TOS) are the common BH3 mimetics belong to class 2 of mitocans. This BH3 mimetics or the agents have shown to interact with the BH3 binding domains and disrupt the interaction of pro-apoptotic (Bax or Bak) and anti-apoptotic (Bcl-2, Bcl-XL or Mcl-1) proteins [70,71,72]. This enhances the channel’s formation in the mitochondrial outer membrane that can release pro-apoptotic molecules such as the cytochrome c and promote apoptosis of the cancer cells.

#### 2.1.3. Class 3 Mitocans: Thiol Redox Inhibitors

The redox environment of cancer cells is distinct from the normal cells, where the cancer cells have higher ROS levels. Therefore, this cancer cell feature makes it more vulnerable to the agents that may target the cancer cells and further enhance oxidative stress in the cancer cells. It is also important to note that the condition of excessive ROS levels or further increase in the ROS levels are toxic for the cells and can also trigger apoptosis. It is well known that cancer cells display higher antioxidant capacity because they adapt to the high level of ROS by activating NF-kB or Nrf2, which increases the expression of antioxidant enzymes [73]. This class of mitocans oxidizes the thiol groups, leading to depletion of the GSH pool in the mitochondria and apoptosis of the cancer cells [74,75]. For example, arsenic trioxide and phenethyl isothiocyanates are class 3 mitocan that have been shown to disturb the normal homeostasis in the cellular redox environment and selectively kill the cancer cells [76,77].

#### 2.1.4. Class 4 Mitocans: VDAC/ANT Targeting Drugs

VDAC is the porin ion channel located in the mitochondria’s outer membrane that allows molecules such as the metabolites, apoptogenic factors, Ca^2+^, and ROS across the mitochondrial membrane. VDAC is found to be overexpressed in cancer cells. The VDAC and ANT togetherly form a permeability transition pore (PTP) complex embedded in the mitochondrial outer membrane and mitochondrial inner membrane. This complex interconnects the mitochondrial matrix with the cellular cytosol and transfers small molecules, including the ADP and ATP [78]. HKII binds to the VDAC and transfers ADP to the ANT1; thus, ANT1 provides ATP to HKII via the VDAC.

Moreover, VDAC also interacts with the anti-apoptotic proteins such as Bcl-2 and Bcl-XL [60]. Therefore, it can be a potential therapeutic intervention in cancer. Thus, the compounds targeting VDAC/ANT1 and inhibiting the binding of VDAC with the anti-apoptotic proteins can be used as a tool to allow apoptosis and fight cancer. Compounds such as lonidamine, arsenites, and steroid analogs are known to modulate the PTP complex that leads to the generation of oxidative stress and induction of apoptosis [79].

#### 2.1.5. Class 5 Mitocans: Electron Transport Chain Targeting Drugs

ATP production in mitochondria is achieved by coordinating electrons from NADH or FADH2 to the ETC complexes (I-V) sequentially. The electrons are carried by the ubiquinone (coenzyme Q) and cytochrome c from complex I to IV. This transfer of electrons generates energy, maintained as the electrochemical proton gradient across the mitochondrial inner membrane. The fifth complex (ATP synthase) utilizes the power generated from the proton gradient to generate ATP from ADP. Though many electrons flow through these complexes, some of the electrons are leaked and generate ROS. ROS’s moderate level is beneficial for cell signaling and survival, but excessive ROS levels are toxic for the cells [80,81]. The deregulated ETC can lead to high ROS generation and shut down vital machinery of cellular metabolism. Based on a similar principle, class 5 mitocans can potentially deregulate ETC complexes and elevate the cancer cells’ ROS levels, leading to cell death. For example, Sorafenib is observed to inhibit ETC complex II, complex III, and ATP synthase in HeLa cells. The dual inhibition of these enzymes further stabilizes and elevates the expression of serine-threonine protein kinase PINK1 on mitochondria’s outer membrane. Sorafenib also triggers the recruitment of the ubiquitin E3 ligase Parkin to damaged mitochondria in these cells. Though PINK1/Parkin is traditionally known to trigger mitophagy in damaged mitochondria, sorafenib treatment triggers PINK1/Parkin-dependent cellular apoptosis. Thus Sorafenib is considered mitocan, and it is also proposed that high Parkin activity levels could make tumor cells more sensitive to sorafenib’s actions [82]. Tamoxifen, which is called MitoTam, has shown an inhibitory effect on the ETC’s complex I, which further caused ROS elevation and cell death [83]. Metformin, an anti-diabetic drug, has also been repurposed for their cancer study and demonstrated a direct effect on the mitochondria by inhibiting the OXPHOS and limiting the citric acid cycle activity in cancer cells [84].

#### 2.1.6. Class 6 mitocans: Lipophilic Cations Targeting the Inner Membrane

It has been observed that the cancer cells have relatively higher net negative transmembrane potential than that of the normal cells, which may be due to the altered bioenergetics of the mitochondria [85]. This feature of the cancer cells makes the lipophilic cations such as Rhodamine-123 and tetraphenylphosphonium ion salts more selective towards the cancer cells. The invention of this class 6 mitocans is based on the Nernst law, which describes that each increase of mitochondrial transmembrane potential by −60 mV corresponds to 10 fold higher accumulation of cationic compounds in the inner mitochondrial membrane [86]. The lipophilic cations penetrate through the hydrophobic barriers of the plasma membrane and mitochondrial membrane and selectively accumulate in the cancer cell’s inner mitochondrial membrane. As a result, the positively charged lipophilic cations reduce the cancer cell mitochondrial membrane’s negative transmembrane potential. The reduced mitochondrial membrane potential may allow various apoptogenic factors like cytochrome c and ROS from the mitochondria and lead to cell death. Moreover, the accumulated lipophilic cations interfere with cells’ physiological function and ultimately trigger apoptosis. (KLAKKLAK)2 is a pro-apoptotic peptide conjugated with penetration that has shown deleterious and selective action on cancer cells by forming a pore in the mitochondrial membrane and establishing significant hydrophobic interactions [87]. Rhodamine -123 and F16 are also delocalized lipophilic cation that selectively accumulates in the mitochondria has shown antiproliferative action towards human gastric carcinoma (SGC-7901) and human breast cancer (MCF-7) cell lines [88].

#### 2.1.7. Class 7 Mitocans: Drugs Targeting the Tricarboxylic Acid Cycle

The Kreb’s cycle (TCA) is the prime source of electrons fed into the ETC. The TCA cycle mechanism starts with converting pyruvate to acetyl-CoA, which is the prerequisite for the entry of pyruvate to the mitochondria and then to the TCA cycle. Pyruvate in the mitochondrial matrix is also converted to oxaloacetate to form citrate or the citric acid, which undergoes a series of reactions and is converted back to oxaloacetate. Further addition of acetyl-CoA to oxaloacetate forms citrate acid, and the cycles go on. During this cycle, the electrons are released and are utilized to drive the electrochemical proton gradient necessary for the ATP generation by OXPHOS. There are specific enzymes known to catalyze Kreb’s cycle’s different reactions, and class 7 mitocans are known to target these enzymes. For instance, pyruvate dehydrogenase converts pyruvate to acetyl-CoA and is regulated by phosphorylation through pyruvate dehydrogenase kinase (PDK). Mitocans such as Dichloroacetate (DCA) selective kills cancer cells by suppressing the PDK activity [89]. Moreover, DCA promotes pyruvate dehydrogenase activity and leads to a metabolic shift from anaerobic to aerobic glycolysis and increases the ROS level. The therapeutic efficiency of DCA is predicted to be dependent on the metabolic profile of cancer cells, and it is also observed that cells with defective mitochondrial metabolism are more sensitive to DCA treatment [89,90]. Similarly, the mitocan 3BP is also known to inhibit the succinate dehydrogenase enzyme, which coverts succinate to fumarate and slow down the TCA cycle [91].

#### 2.1.8. Class 8 Mitocans: Drugs Targeting mtDNA

The size of mammalian mtDNA is about 16 kB, and it encodes the 13 subunits of the four ETC complexes, 24 tRNAs, 12S and 16S rRNA. It also consists of a region called the D-loop domain that plays a vital role in regulating mtDNA replication [92]. Class 8 mitocans are known to target the mtDNA of the cancer cells by interfering with the mtDNA stability and affecting the activity of polymerase-γ that is specific for replicating mtDNA. For example, vitamin K3 or menadione is the mitocan that targets the mtDNA by inhibiting the activity of mtDNA polymerase-γ, thereby inducing apoptosis of the cancer cells [93]. Another mitocan is the 1-methyl-4-phenyl-pyridinium that directly destabilized the mtDNA’s D-loop structure in HeLa S3 cells [94]. Rhodocyanine dye MKT-077 selectively induced mtDNA damage in the CX-l cell line (human colon carcinoma) and also inhibits mitochondrial respiration, indicating MKT-007 as an effective metabolic inhibitor [95,96].

Thus the different classes of mitocans are proposed to target other proteins/pathways, which may eventually affect the mitochondrial metabolism in cancer cells. In a similar context, the upcoming significance of mutant p53 in regulating glucose metabolism and Zn deficiency in disturbing the cellular redox should not be overlooked. The use of mitocans may be applicable in such conditions. p53 is thought to suppress tumorigenesis primarily by inducing cell cycle arrest, apoptosis, and senescence in response to stress. However, recent studies have also revealed the non-canonical functions of p53 in the regulation of glycolysis, pentose phosphate pathway, mitochondrial oxidative phosphorylation, and lipid metabolism, which contributes to the role of p53 in tumor progression [97,98]. p53 is mutated in about half of cancers, and mutant p53 promotes adaptive responses to cancer-related stress conditions to support tumor progression [97]. Studies indicate that mutant p53 up-regulates the glucose metabolism and OXPHOS in cancer cells [99,100] and thus shown to sustain anabolic growth by enhancing glucose import and promoting the Warburg effect mutant p53 knock-in mice [101]. Anticancer therapies based on drug combinations that either directly or indirectly hit mutant p53-dependent homeostatic circuits can be expected to provide several exciting therapeutic possibilities. To break such circuits, the use of mitocans can be a promising approach.

Zinc is a redox-inactive metal and crucial for p53 activity in normal cells [102]. Zn is an important component of the antioxidant network, and growing pieces of evidence indicate its involvement in redox-regulated signaling [103,104]. Though the mechanisms through which Zn contributes to balancing the redox are still partially known and the subject of active research. Most commonly, the Zn deficiency is known to disrupt p53 function [105] and disturb the cellular redox in esophageal, head, and neck cancer cells [106,107,108,109,110]

As discussed above, different classes of mitocans are known to target glucose metabolism (class 1), redox imbalance (class 3), and ETC (class 5) in cancer cells. Hence the cancer cells, which are p53 mutant (the most common cause) and Zn deficient, can also be targeted using these specific classes of mitocans.

### 2.2. The Evolution and Current Status of Mitocans

After the introduction of mitocans it significantly widened the hunt for potential mitocans. The study associated with mitocans is still under research, but there is enough evidence to support the mitochondria-targeting the ability of various synthetic and natural compounds. From the beginning till date, many combinations can be counted under the umbrella of mitocans. Most of the recorded mitocans are artificial, and they are either in different phases of clinical trials or in preclinical studies. Many of these compounds also reached different clinical trial stages but failed to get approved as a drug and terminated from the trials [111]. The main reason for their termination from the study was their low sensitivity and efficacy, which was reported to be the significant limitations associated with different synthetic mitocans studied so far. For example, mitocans like 1-methyl-4-phenyl-pyridinium, 2DG, vitamin K3, DCA, and F16 were terminated from the clinical trial. These mitocans failed to prove themselves in terms of sensitivity and efficacy in the latter stage of clinical trials. On the other side, Mito-LND and NSC13062 are the compounds that have the preliminary evidence of affecting the mitochondria of cancer cells and are proposed as possible mitocans [112,113,114,115,116]. Table 1a,b enlist some widely studied synthetic mitocans, their class, and associated studies. Despite so much trial and studies, we do not have any ready to use mitocan, but the attempts are still ongoing. Perhaps, these compounds require more experimental pieces of evidence to proceed for the clinical trials.

## 3. Natural Agents as Mitocans: The Alternative Approach to Overcome the Limitations of Synthetic Mitocans

The failures of these synthetic compounds necessitated focusing on improving the drawbacks of enlisted mitocans. Given their constraints, the research in the past few years is also trying to explore the various analogs and the derivatives of mitocans. On the verge of finding improvised mitocans, it was proposed to utilize natural sources to formulate mitocans [39]. There is no doubt that the folklore medicines used in ancient times have left an impeccable record of healing several diseases [40]. These ancient folklore medicines are known to be more organic. There is no doubt that ancient age has always relied on herbal medicines and lived longer with minimal health issues. The centuries-old practice of natural remedies is still followed in the present Ayurveda and Unani treatments, which are well known for their significant and long-lasting results [149,150,151]. This has encouraged the current research to utilize the folklore medicinal herbs to formulate naturally derived drugs and redirect the scientific fraternity towards the formulation of natural mitocans (i.e., mitocans derived from the natural medicinal herbs). The search for natural mitocans has begun. A good number of natural agents are known to exhibit the anticancer property. Based upon their ability to affect the mitochondrial function (either directly or indirectly) in cancer cells, they may be suggested to behave as potential mitocans. Apart from pure natural compounds, different plant-derived crude extracts are also reported to exhibit mitochondria-targeting ability in cancer cells.

### 3.1. Classification of Natural Agents and Plant Extracts as Mitocans

Different plant-based bioactive compounds are proven to exhibit their cytotoxic activity by targeting the mitochondria of cancer cells. However, their respective mitocan classes are not yet defined. Very few natural agents like phenethyl isothiocyanates, benzyl isothiocyanate, and gossypol have been categorized under different classes of mitocans. For instance, isothiocyanates are known to increase the ROS level in human leukemia cells in vitro and, in turn, trigger apoptosis in these cells [152]. On the other hand, gossypol is known to act as a pro-apoptotic protein by inhibiting the Bcl-2 family protein observed in leukemic cells [70,153] Based on their mode of action, these two compounds have been classified under class 3 and 2 of the mitocan class. In the following section of the review, we have summarized all the reported natural agents and their known action mechanisms. After reviewing their detailed anticancer activities, we have further categorized these natural agents under suitable mitocan classes and put forth a possible explanation for the same (Table 2). 

In addition to studies on isolated natural agents, there is a plethora of information regarding different herbal crude extracts and their ability to inhibit mitochondrial activity in cancer cells. These natural extracts have also been suggested to be possible mitocans by different in vitro and in vivo studies based upon their biological activities. As they have not been categorized into other mitocan classes, our review’s illustrative purpose is to bring together all such unclassified mitocans and categorize them under various mitocans (Table 3). After analyzing all the studies regarding natural agents and plant extracts, we interestingly observed that most of these compounds belong to the class 1 (inhibitors of HKII), class 2 (compounds targeting Bcl-2 family proteins), class 3 (compounds elevating the ROS level), and class 6 (lipophilic cations targeting inner membrane) (Table 2 and Table 3).

### 3.2. Natural Agents which Inhibit HKII 

Glucose is the first substrate for cellular respiration, and it enters the cells via different types of glucose transporters (GLUT1–GLUT5), depending on the cell types [54,55]. To make it accessible in the form of energy, the glucose has to be broken down. To generate energy out of glucose, it undergoes glycolysis, which is the first cellular respiration pathway that oxidizes glucose molecules and produces pyruvic acid (pyruvate), ATP, and reduced NADH. The very first enzyme, or the critical mediator of aerobic glycolysis, is the HKII [190]. In normal cells, the incoming glucose and ATP as another substrate binds to HKII, leading to the production of G6P, which is distributed for the glycolysis and hexose monophosphate (HMP) shunting [191]. HMP shunting is associated with two major functions, i.e., generating nicotinamide adenine dinucleotide phosphate hydrogen (NADPH) and providing ribose 5-phosphate (R5P). However, there are individual differences observed in the cancer cell environment. It starts with the overexpression of HKII in cancer cells, and hence, the rate of glucose uptake is also higher [55]. The high rate of glycolysis may lead to excessive production of ATP and lactate in these cells. Increased ATP amounts may help in cell proliferation, and higher amounts of lactate would be exported outside these cells by suitable transporter proteins (as explained above). The higher lactate level reduces the extracellular pH that, in turn, helps in the migration and invasion of cancer cells.

Due to higher expression and activity of HKII, the production of R5P via HMP shunting is also increased and thus helps in nucleic acid synthesis in cancer cells. Higher HKII also increases the NADPH level, which in turn reduces the susceptibility towards ROS. Thus, inhibition of HKII can exhibit a cytostatic effect on the cancer cells by reducing ATP production and DNA replication. It can also demonstrate a cytotoxic effect by increasing the susceptibility towards ROS and mitochondria-mediated apoptosis in cancer cells. Therefore, inhibition of HKII is one of the important aspects of cancer therapeutics.

It was observed that the natural agents methyl jasmonate (MJ) and curcumin exhibited cytotoxicity towards cancer cells by targeting the HKII. Studies of MJ in four different cell types (murine melanoma: B16, murine colon carcinoma: CT26, murine B cell leukemia: BCL1, human T lymphoblastic leukemia cell line: Molt-4) revealed that MJ at a dose of 3 mM and incubation period of 30 minutes dissociates HKII from VDAC and, as a result, inhibits the activity of HKII [172]. Apart from these in vitro models, different in vivo studies have used both mM and uM concentrations of MJ in various animal models. As a result, MJ was observed to exhibit the antiproliferative effect and increased survival of animals. Interestingly, none of the studies report any significant toxicity in normal cells [173,192,193,194,195,196,197]

**Figure 3:**
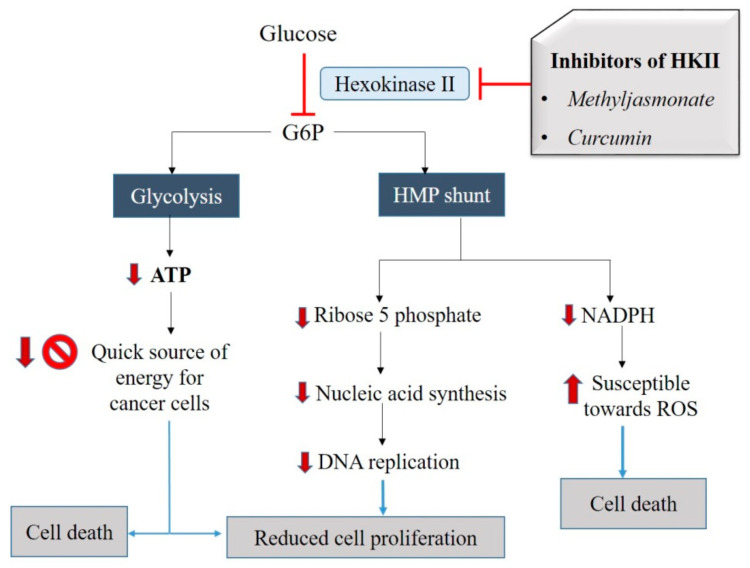
Schematic representation of the inhibitory effect of herbal mitocans on HKII. Inhibition of HKII may inhibit the conversion of glucose to G6P and interfere with the process of glycolysis as well as HMP shunt, leading to the cytostatic/cytotoxic effect on cancer cells. Methyl jasmonate and curcumin can be cytostatic by reducing cell proliferation and cytotoxic by killing the cancer cells.

Similarly, Wang et al. (2015) performed a study of human colorectal cancer cells (HCT116 and HT29) which were treated with varying concentrations (10, 20, and 40 μmol/L) of curcumin and incubated for 24 hrs. It was observed that HKII detaches from the mitochondria of the curcumin-treated HCT116 and HT29 cells [168]. Therefore, these studies suggest that curcumin and MJ enhanced dissociation of HKII from mitochondria and hence inhibited glucose uptake and lactate generation, causing inhibition of cancer cell energy metabolism and triggering cell death (Figure 3). Therefore, based on these suggested effects of MJ and curcumin on the activity of HKII, we may propose these compounds to belong to class 1 of mitocans.

### 3.3. Natural Agents Targeting Bcl-2 Family Proteins

Bcl-2 family proteins are prominently essential gatekeepers to the apoptotic response in the cells. This family of proteins is composed of three groups: the anti-apoptosis proteins (Bcl-2, Bcl-XL, Bcl-W), pro-apoptotic effectors (Bax, Bak, and BOK), and pro-apoptotic activators (BH3-only proteins: BAD, BAD, BIK, BIM, BMF, HRK, NOXA, PUMA, etc.) [61]. These three groups of the Bcl-2 family proteins function via different mechanisms of actions to regulate the apoptosis process of the cell. In response to apoptosis stimulus (cellular stress, i.e., damage to the lipids, proteins, DNA, and RNA), the pro-apoptosis proteins are activated. Upon activation, BH3-only proteins form a hydrophobic groove and bind to the Bcl-2 proteins, whose hydrophobic carboxyl-terminal domain is linked to the mitochondrial outer membrane. Henceforth, this binding of BH3-only proteins inhibits the anti-apoptosis action of the Bcl-2 proteins. Additionally, the other pro-apoptosis proteins Bax and Bak in their active form are known to oligomerize in the form of homo-/hetero-oligomers. These oligomers of Bax or Bak form a pore-like structure that permeabilizes the mitochondrial outer membrane to execute several apoptogenic molecules from the mitochondrion. Apoptogenic molecules such as cytochrome c activate the apoptotic protease-activating factor-1 (Apaf-1), which in its activated form binds to the caspase-9 to form the apoptosome complex. This complex activates caspase-3, triggering the activation of different executioner caspases and complete cell death [34]. Over the past years, cancer therapeutics have been conceptualized to either initiate the pro-apoptotic proteins or inhibit anti-apoptotic proteins in killing the cancer cell [70,71,72,87]. Different experimental studies revealed that flavokawain A, berberine, asiatic acid, and andrographolide are the natural agents that induce apoptosis mediated via the action of Bcl-2 family proteins.

Flavokawain A is the bioactive compound of *Piper methysticum*, and its antiproliferative activity has been studied in different cancer cells. The research performed on breast cancer cells (MCF-7 and MDA-MB231) suggested that flavokawain A increased the expression of Bax and the change in the mitochondrial membrane potential. These changes consequently lead to the secretion of cytochrome c that activates the caspase cascade and causes cell death in these cells [170]. Another study on bladder cancer cells (T24 cells) and bladder tumor cells induced in a mice model also revealed an increase in active Bax protein. Moreover, this study also observed compromised levels of Bcl-XL expression and a further decrease in the association of Bcl-XL to Bax.

As a consequence, the active form of Bax proteins was also increased. The activated Bax protein resulted in the loss of mitochondrial membrane potential that led to the release of cytochrome c, ultimately causing apoptosis [171]. The anti-cancer property of Berberine, a bioactive compound of *Coptidis rhizoma*, was also studied in breast cancer cells (MCF-7), liver cancer cells (HUH-7), and oral cancer cells (HSC). As a result, it was evident that berberine exhibited a similar anti-cancer mechanism like flavokawain A [163,164,165]. Additionally, the impact of berberine studied in normal breast cancer cells (MCF-12F) revealed their non-toxicity towards the normal breast cells [164]. 

Asiatic acid, the bioactive compound of *Centella asiatica,* was studied in different in vitro and in vivo model systems. The results of various studies suggested that asiatic acid triggers the mitochondria-mediated apoptosis in cancer cells. Yet, the detailed molecular mechanism for its anti-cancer nature is distinct in different cancer cell types. For example, asiatic acid studied in human melanoma cells (SK-MEL-2) showed an increased level of ROS and hence increased the expression of pro-apoptotic Bax protein, without affecting the expression level of Bcl-2 protein. Due to differential effects on both these proteins’ expression, the Bax/Bcl-2 ratio was increased, which eventually led to apoptotic cell death via triggering the activation of a cascade of caspases [160]. Another study by Tang et al. (2009) suggests that asiatic acid induces loss of MMP and releases cytochrome c, which further activates the caspase activity and poly (ADP-ribose) polymerase (PARP) cleavage resulting in apoptotic death in the tumor cells [161]. The effect of asiatic acid was also studied on human lung cancer cell lines (A549 and H1299) and tumor-induced mice model. Both in vitro and in vivo studies demonstrated that there was a loss of mitochondrial membrane integrity leading to generation of ROS and mitochondria-mediated cell death [162]. 

Andrographolide isolated from *Andrographis paniculata* is reported to induce cell cycle arrest in the G0/G1 phase and mitochondria-mediated cell death in human leukemic HL-60 cells [157]. This arrest in the cell cycle was found to be correlated with altered expression of Bax and Bcl-2 proteins and cell death [157]. Interestingly, a study conducted by Yang et al. further highlighted that andrographolide treatment significantly altered Bax proteins’ conformation in hepatocellular carcinoma (SMMC-7721) cells [158]. Apart from its usual effect on pro- and anti-apoptotic protein expression, andrographolide was also observed to increase the ROS level in colon cancer cells (T84 and COLO 205) [159]. 

Curcumin was also studied in different cell lines (RS4;11 and SupB1) of B-precursor acute lymphoblastic leukemia. The results suggest curcumin’s involvement in increasing Bax expression and decreasing the Bcl-2 proteins in treated cells. Consequently, it resulted in disturbance to mitochondrial membrane permeabilization and led to the loss of mitochondrial membrane potential. The reduced mitochondrial membrane potential induces the intrinsic pathway of apoptosis. Further investigations revealed a dose-dependent generation of ROS and further emphasized the role of ROS levels in the induction of apoptosis in cancer cells [169].

Based upon the above-discussed mitochondria-targeting the ability of asiatic acid, andrographolide, berberine flavokawain A, and curcumin in cancer cells and their comparison with reported mitocans, we propose that all these five compounds may behave as potential mitocans and, specifically, belong to class 2 of mitocans. Though all these compounds were observed to exhibit their anticancer property by similar mechanisms, asiatic acid, andrographolide, and curcumin also elevated ROS levels. They triggered apoptosis in lung cancer cells (A549 and H1299), colon cancer cells (T84 and COLO 205), and B-precursor acute lymphoblastic leukemia cells (697, REH, RS4;11, and SupB15) respectively. These observations may suggest their role as the elevators of ROS, as well as Bax/Bcl-2 ratio. Hence, because of the uniqueness of asiatic acid, andrographolide, and curcumin as ROS elevators, these three compounds may be categorized under class 3 of mitocans, as well. 

**Figure 4:**
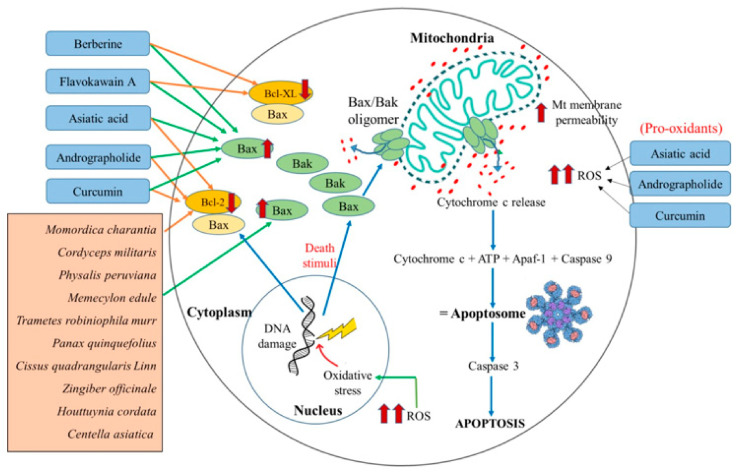
Schematic representation of natural agents’ role inducing apoptosis via increasing the level of pro-apoptotic Bax and decreasing the level of anti-apoptotic Bcl-2/Bcl-XL. Death stimuli due to damage in the DNA activates Bax. Activated Bax forms a homo/heterooligomer, whose pore-like structure channelizes the release of cytochrome c. The mitochondrial membrane permeability increases, which, in turn, allows increased cytochrome c release. Cytochrome c, ATP, Apaf-1, and caspase-9 form an apoptosome complex that further activates caspase-3 and leads to apoptosis. Possible natural mitocans flavokawain A, berberine downregulated the Bcl-XL and upregulated the Bax. On the other hand, asiatic acid and andrographolide decreased the expression of Bcl-2 and increased the expression of Bax and the ROS level. Crude extracts of *Momordica charantia, Cordyceps militaris, Physalis peruviana, Memecylon edule, Trametes robiniophila murr, Panax quinquefolius, Cissus quadrangularis Linn, Zingiber officinale, Houttuynia cordata,* and *Centella asiatica* increased the expression of Bax and decreased the expression of Bcl-2 proteins.

As per Table 3, we also reviewed and observed that most crude extracts of anti-cancer herbs reported to target the mitochondria of cancer cells were influencers of Bcl-2 family proteins. The anti-cancer effect of various natural extracts of the following eight herbs, *Momordica charantia, Cordyceps militaris, Physalis peruviana, Memecylon edule, Trametes robiniophila murr, Panax quinquefolius, Cissus quadrangularis Linn*, *Zingiber officinale*, *Houttuynia cordata*, and *Centella asiatica,* were studied on different cancer cell lines such as the human nasopharyngeal carcinoma cells (Hone-1), gastric adenocarcinoma cells (AGS), colorectal carcinoma cells (HCT-116), lung adenocarcinoma cell (CL1-0), human breast cancer cell lines (MCF-7 and MDA-MB-231), human liver cancer cells (Hep G2), gastric cancer cells (MKN-74), human skin epidermoid carcinoma (A431), human colon adenocarcinoma (HT-29), and prostate cancer cells (PC3). These studies suggested that the crude extracts of all these anti-cancer herbs shared the common mechanism of actions. All these natural extracts significantly increased the expression of Bax protein and also decreased the level of Bcl-2 protein in individual cancer cells. Consequently, the Bax/Bcl-2 ratio increases, and the mitochondrial membrane’s permeability increases [169,176,177,178,179,183,184]. The released cytochrome c in the cytosol further induces the signal of cell death by intrinsic pathway. The schematic representation of the illustrated mechanism in cancer cells is shown in Figure 4.

### 3.4. Natural Agents as ROS Elevators

ROS is a byproduct of OXPHOS, and its higher level may cause oxidative damage to different biomolecules such as protein, lipids, carbohydrates, and DNA, too. ROS is predominantly generated in mitochondria, and in higher concentrations it may directly affect the biomolecules present in its closest proximity. The elevated levels of ROS are known to damage the mitochondrial membrane, as well as mtDNA eventually. ROS in higher concentrations is known to exhibit a damaging effect to the cell; however, at a lower concentration, ROS is known to act as a potential mitogen, promote cell division, improve cell viability, and help in cellular differentiation via different cell signaling pathways such as MAPK, PI3-AKT, NF-kappa B, and Nrf-2 [198]. The redox environment of cancer cells is distinct from that of normal cells. To keep up with their uncontrolled rate of proliferation and higher energy demands, cancer cells have an elevated metabolic rate.

Consequently, it may lead to respiratory dysfunctions and compromised coupling efficiency of ETC, which may increase the electron leakage, causing higher ROS levels. Increased ROS levels make the cancer cells more vulnerable towards the agents that can further raise oxidative stress. The damage caused due to the oxidative stress contributes to alterations of proteins via the reaction between the protein amino acid residues and the ROS. For instance, the cysteine residues of proteins (which generally exist in thiolate form) undergo ROS oxidative modification. At higher levels of ROS, the oxidized thiolate anions form a variety of cysteine oxidation products, which includes disulfide formation (S-S-), S-glutathionylation (protein-SSG), S-nitrosylation (-SNO), sulfenic acid formation (-SOH), which play important roles in redox regulation of protein functions by ROS [199]. These oxidation products may protect the target proteins from further oxidation that can permanently damage the target proteins. Hence, such oxidative modifications can enhance the protein functioning and signal for cell survival and proliferation in cancer cells. Thus, the elevated levels of ROS may boost oxidative modifications and encourage survival and the further vigorous proliferation of cancer cells. In cancer, however, the ROS level is comparatively much higher, but the further increase may trigger oxidative damage to these cells [200]. Such compounds which increase the ROS levels are known as “pro-oxidants” [201]. Therefore, further elevating the levels of ROS may be used as an important aspect of cancer therapeutics. It was observed that the natural agent bezeille extracted from *Scutellaria barbata* has the potential to target mitochondria of the cancer cell by elevating their ROS level that may, in turn, inhibit their glycolysis OXPHOS. Inhibition of these processes may bring the cell to a lower energy state and further lead to their apoptotic phase. Experimental evidence suggests that bezeille (ROS elevator) may trigger the apoptosis of breast cancer cells (MDAMB231) in three different ways [167]. Firstly, an elevated level of ROS may cause DNA damage due to oxidative stress. Subsequently, in response to DNA damage, the poly (ADP-ribose) polymerase (PARP), a repair enzyme for DNA damage, gets activated. As NAD^+^ is a known substrate for PARP, hence with excessive activation of PARP, the NAD^+^ levels were observed to reduce in MDAMB231 cells. 

Subsequently, in response to excessive DNA damage, the poly (ADP-ribose) polymerase (PARP), a repair enzyme for DNA damage, gets massively activated. The activated PARP was observed to be well-correlated with a low level of both NAD^+^ (as PARP consumes NAD^+^) and ATP in MDAMB231 cells. In case of excessive activation of PARP, when the substrate NAD^+^ is exhausted, both the electron transfer in mitochondria (ETC) and glycolysis cannot take place, and thus, ATP levels may go down. 

Secondly, oxidative damage due to higher ROS levels may also deplete the NADPH required for the oxidation of GSSG to GSH and hence decrease the GSH/GSSG ratio important for maintaining the redox environment of the cell. Therefore, NADPH and GSH depletion may collapse the redox balance of the cell. Thirdly, elevated ROS levels may also interfere in the electron transfer in OXPHOS. Interference in the OXPHOS causes leakage of electrons from the ETC. It increases the mitochondrial membrane permeability that may help release cytochrome c to the cytosol and trigger programmed cell death through apoptosis. Altogether, these three mechanisms observed by Chen et al. suggest that bezielle elevates the ROS level and induces redox imbalance in the cancer cell environment [167]. 

**Figure 5:**
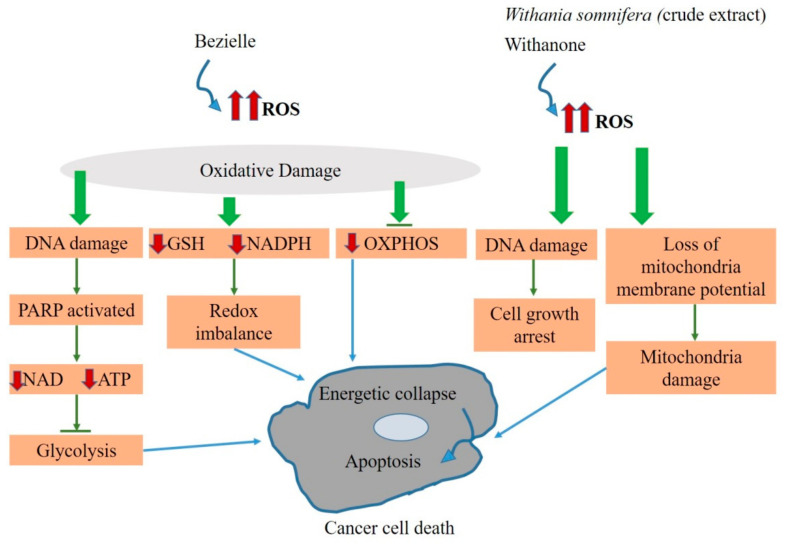
Schematic representation of the anti-cancer effects of some selected natural agents or extracts by influencing the ROS levels in cancer cells. Bezielle elevates the level of ROS in the cancer cell and causes redox imbalance, as well as inhibiting glycolysis and OXPHOS. Similarly, the methanolic extract of *Withania somnifera* and its bioactive compound withanone also elevated the ROS level in the cancer cell, causing cell growth arrest and mitochondria damage. As a consequence of redox imbalance, inhibition of the energy-producing pathways (glycolysis and OXPHOS) and mitochondrial damage may cause energetic collapse and apoptosis.

The crude extract of *Withania somnifera* leaves and its bioactive compound withanone was also studied in breast cancer cells (MCF-7) and colon cancer cells (HCT116). As a result, both withanone and the methanolic leaves extract of *Withania somnifera* were observed to behave as ROS-producing agents. The suggested mechanism of action explains that the withanone- and *Withania somnifera*-induced ROS signaling may cause DNA damage and lead to cell growth arrest in MCF-7 and HCT116 cells. Moreover, it may also cause a loss of mitochondrial membrane potential and lead to mitochondrial damage, causing cell death [174]. Based on evidence-based studies, bezeille, withanone, and crude extract of *Withania somnifera* can be distributed under class 3 of mitocans which are explained to elevate the level of oxidative stress, affecting the equilibrium of cellular redox, a significant factor responsible for inducing mitochondria-mediated cell death in cancer cells (Figure 5). 

### 3.5. Natural Agents Reducing the Mitochondrial Membrane Potential

Mitochondria are double membraned organelles that are selective towards the permeability of certain compounds that may move in and out of the mitochondria. The outer membrane of the mitochondria significantly regulates various receptor enzymes that are important for the regulatory signals associated with cell survival and death. Upon oxidative stress and activation of the pro-apoptotic proteins, the mitochondrial membrane porosity increases and hence permeabilizes the excessive escape of cytochrome c to the cytosol, leading to cell death. On the other hand, the inner mitochondrial membrane is the locus for the OXPHOS. The process of aerobic respiration via ETC demonstrates the sequential transfer of electrons through the enzyme complexes I, II, III, and IV of the respiratory chain [202]. The movement of electrons through these four complexes produces energy, which is utilized to pump protons from the mitochondrial matrix to its intermembrane space. However, the pumped protons in the intermembrane area cannot move out because the mitochondrial membrane does not allow the passage of ions. As a result, it generates an electrochemical gradient where the intermembrane space holds a higher concentration of H^+^ ions, and the matrix contains the lower concentration of H^+^ ions. This electrochemical gradient causes the mitochondrial membrane potential. It drives the pumping back of H^+^ ions from intermembrane space to its matrix through the enzyme ATP synthase that synthesizes ATP from ADP and phosphate (Pi) [203]. Altogether, the cells must maintain a hyperpolarized voltage across their inner mitochondrial membranes. If this hyperpolarization dissipates, the voltage-sensitive PTP will open and release pro-apoptotic agents (e.g., cytochrome c) into the cytoplasm and lead to apoptotic cell death [30].

In comparison to normal cells, cancer cells have a higher metabolic rate. The higher energy demand by cancer cells leads to the vigorous activity of ETC that, in turn, increases the pumping of H^+^ ions into the intermembrane space and hence increases the electrochemical gradient. This increase in the electrochemical gradient subsequently leads to hyperpolarized mitochondrial membrane potential than non-malignant cells. The hyperpolarization of mitochondrial membrane potential can be >50% greater in cancer cells than in normal cells. Hence, it serves as the hallmark of cancer cells that can be selectively targeted in cancer cells. The mitocans of class 6 are lipophilic cations that significantly target the inner membrane of the mitochondria. However, our review on the possible herbal mitocans observed that the xanthones that are the bioactive compound of *Garcinia mangostana* led to the loss of mitochondrial membrane potential of human colorectal carcinoma cells (HCT 116) and human leukemia cells (HL60) [175,176]. Similarly, there are also different crude extracts of the anticancer herbs *Centella asiatica*, *Houttuynia cordata Thunb, Cinnamomum cassia,* and *Gracilaria tenuistipitata* that are reported to cause loss of mitochondrial membrane potential of human cervical cancer (SiHa), mouse hepatoma cells (H22), oral squamous cell cancer (Ca9-22), human colon adenocarcinoma cells (HT-29), and breast cancer cells (MCF-7), respectively [181,182,184,188]. This loss of mitochondrial membrane potential permeabilizes the release of various apoptogenic factors such as cytochrome c, which further leads to apoptosis of the cancer cells. However, it is not yet clear about the mechanism of whether the observed loss of mitochondrial membrane potential is for the inner membrane or the outer membrane of the mitochondria.

## 4. Conclusions and Future Perspectives

In conclusion, we summarize and highlight the abundance of an existing natural agent with their cancer cell-targeting ability through a unique mechanism of damaging mitochondria. After comparing the mode of action of all these natural agents, we observed that the majority of our reviewed natural agents reduce the expression of Bcl-2 protein (anti-apoptotic) and/or increase the level of Bax proteins (pro-apoptotic) in target cells. Thus, most of these natural agents may be hypothesized to belong to class 2 mitocans, although it may be primitive to draw any such conclusion. The bioenergetics of normal cells and cancer cells are dramatically different. Hence, we suggest that all-natural agents showing deleterious effects on mitochondrial health and its metabolism can be selected and studied for their cytotoxic effect on cancer cells using various approaches, including in vitro and in vivo model systems. After confirming their impact on the cancer cells, their doses, formulations, and modes of delivery can be optimized to improve efficacy and compromised side effects (if any) on the neighboring healthy cells. Our proposed study attempts to design alternative cancer therapy using natural sources carrying mitochondria-targeting ability. It may be proven as a promising and safer mode of cancer treatment in the future. 

These mitocans are specific in terms of targeting the defective mitochondria of cancer cells, hence, to provide a broader future application of such agents, it may be proposed that these mitocans can also be used in other diseases such as neurodegenerative disorders (NDDs), which share the metabolic alterations, increased ROS, mitochondria-related dysfunctions, and altered regulation of cell survival or death. Both Alzheimer’s and Parkinson’s diseases are less frequent in survivors of many cancers (and vice versa), suggesting that a tendency towards one family of diseases may decrease the risk of the other [204,205,206,207,208]. Studies also revealed that p53, the major checkpoints protein, is at the crossroad between cancer and dementia. The misfolded p53 is considered to be an early indicator of dementia [209]. In most of the NDDs, including dementia, the deregulation of mitochondrial functions leads to high ROS production, eventually causing altered signaling of the apoptotic mechanisms and cell death of neurons. As NDDs, together with cancer, represents a burden for both caregivers and society, and mitochondrial dysfunction is a key factor in the pathogenesis of NDDs, the compounds targeting mitochondria of neuronal cells should also be investigated. However, the aim to use mitochondria-targeting drugs in neuronal cells should be entirely different from cancer. As mitocans aim to induce mitochondria-mediated apoptosis in cancer cells, the compound targeting mitochondria of neurons should aim to improve the metabolism and prevent neuronal cells from apoptosis. Hence, the mitochondria-targeting compound in NDDs could aim to trigger selective autophagy of damaged mitochondria (mitophagy) in neurons, which is known to be impaired in most of the NDD-related cases. 

## Figures and Tables

**Table 1:** (a) List of synthetic mitocans and their current status in clinical trials. (b) List of synthetic mitocans, and their current status in preclinical and in vitro studies.

(a)					
S. No.	Synthetic Compound	Mode of Action	Mitocan Class *	Current Status	References
1.	ABT-263 (Navitoclax)	Inhibits anti-apoptotic Bcl-2 family proteins (Bcl-XL, Bcl-2, and Bcl-w). Induces translocation of Bax, release of cytochrome c.	2	Phase II of the clinical trial	[71,117,118]
2.	1-methyl-4-phenyl-pyridinium	Affects the overall function of mitochondria by inhibiting ATP generation	8	Failed to cross the blood-brain barrier	[119]
3.	Vitamin K3 or Menadione	Inhibits DNA polymerase γ and increased ROS generation	8	Failed in phase II of the clinical trial (https://clinicaltrials.gov)	[93,120]
4.	Dichloroacetate, (DCA)	Unbalance the redox homeostasis and overproduction of ROS	7	Terminated from phase II clinical trial (https://clinicaltrials.gov	[90,121]
5.	Mito-Tam (a mitochondrial-targeted derivative of tamoxifen.)	Inhibits complex I- driven respiration	5	Phase I of the clinical trial	[83,122]
6.	α-TOS	Targets Complex II and accumulation of ROS	5 & 2	Completed Phase III of clinical trial	[123,124,125]
7.	2-Deoxy-D-glucose (2DG)	Increased levels of glucose transporter expression and glucose uptake. Inhibits hexokinase and hexose phosphate isomerase	1	Terminated after phase I clinical trial Study for combination with other drugs.	[64,126,127]
8.	F16	Accumulation in cancer cell mitochondria leading to apoptosis	6	Terminated from phase II clinical trial (https://clinicaltrials.gov)	[88,128,129]
9.	Arsenic trioxide	Induces oxidative stress, DNA damage, change in mitochondrial membrane potential, translocation, and upregulation of apoptotic proteins	3	Phase II of a clinical trial (https://clinicaltrials.gov)	[76,130,131]
10.	Benserazide	Reduce the uptake of glucose, production of lactate, level of ATP and causes apoptosis	1	Phase IV of the clinical trial	[65]
11.	Metformin	Target mitochondrial ETC; Exerts oxidative stress	5	Phase I of clinical trials	[83,84,132]
12.	Sorafenib	Inhibition of ATP synthase	5	Phase III of the clinical trial	[82]
**(b)**					
**S. No.**	**Synthetic Compound**	**Mode of action**	Mitocan Class *	**Current status**	**References**
1.	Antimycin A	Inhibits succinate, nicotinamide adenine dinucleotide (NADH) oxidase, and electron transport between cytochrome b & c	2 & 5	Preclinical studies	[133,134]
2.	MKT-007	Targets mtDNA, a metabolic inhibitor	8	Preclinical studies and in vitro studies	[96,135]
3.	Adaphostin	Inhibits complex II and III of ETC and accumulation of ROS	5	Preclinical stage and combinatorial study with other drugs.	[136,137,138]
4.	3 Bromopyruvate (3BP)	Inhibition of HK II, glyceraldehyde-3-phosphate dehydrogenase and LDH, Induces the mitochondrial protein leakage and block the electron transport system	1 & 5	Under research to improve its specificity, sensitivity & efficacy by combining with other anticancer agents or formulating with targeted liposomes to enhance its delivery.	[63,139,140]
5.	Rhodamine-123	Accumulation in mitochondria	6	No current update as a mitocan	[85,141]
6.	(KLAKKLAK)2 (Pro-apoptotic Peptide)	Disrupt mitochondrial membrane	6	Understudy for improving its efficacy	[87,142,143]
7.	Mito-LND	Inhibition of mitochondrial complexes I and II, and stimulation of ROS production	5	Pre-clinical studies	[112,114,115]
8.	Danshensu-Tetramethylpyrazine (DT-010)	Inhibition of mitochondrial complex II	7	Preclinical studies	[144,145]
9.	NSC13062	Target mitochondrial ETC	5	Preclinical studies and in vitro studies	[113,116]
10.	Mito-K3 (derivative of Menadione)	Accumulates in mitochondria, interferes with redox property, and causes mitochondrial dysfunction	8	Preclinical studies	[146]
11.	MitoVE11S (CNC332)	Targets mitochondrial complex II	5 & 8	Preclinical and in vitro studies	[50,147,148]

* For all the compounds in Table 1a and 1–6 compound of Table 1b, the mitocan class has already been justified and published. However, for the rest of the compounds (7–11) in Table 1b, we have proposed the suitable mitocan class based on their known molecular mechanism.

**Table 2:** Categorization of natural agents into different classes of mitocans.

S. No.	Natural Agent (Source)	Mode of action	Mitocan Class	Current status	References
1.	Phenethyl isothiocyanates (cruciferous vegetables)	Induction of oxidative stress and triggering of Ca^2+^ flux, which leads to mitochondrial cell death mechanisms	3	Phase I clinical trial (https://clinicaltrials.gov)	[154,155,156]
2.	Benzyl isothiocyanate (brassicas)	Intrinsic apoptosis is mediated via ROS production and mitochondrial dysfunction.	3	In vitro studies going on	[154,155]
3.	Gossypol (cotton plant)	Inhibits Bcl-2, Bcl-XL, Bcl-W, Mcl-1	2	Phase II of the clinical trial (https://clinicaltrials.gov)	[70,153]
4.	Andrographolide (*Andrographis paniculata)*	Targets Bcl-2 family protein and cyclophilin D; Increased ROS production	2	In vitro study	[157,158,159]
5.	Asiatic acid *(Centella asiatica)*	Increases mitochondria membrane permeability, ROS generation, alteration of Bax/Bcl-2 ratio, and activation of caspase-3	2	In vitro study	[160,161,162]
6.	Berberine *(Coptidis rhizoma)*	Increases expression of Bax; Decreases Bcl-2 expression level; Induces ROS and Ca^2+^ production; Loss of mitochondria membrane permeability	2	In vitro study	[163,164,165]
7.	Bezielle (*Scutellaria barbata*)	Inhibits Glycolysis and OXPHOS by increasing the ROS level	3	In vitro study	[166,167]
8.	Curcumin (*Curcuma longa*)	Downregulation of expression and activity of HK II; Loss of mitochondria membrane potential	1 and 2	In vitro study	[168,169]
9.	Flavokawain A (*Piper methysticum*)	Induces mitochondrial-dependent apoptosis by increasing the expression of Bax	2	In vitro and in vivo study	[170,171]
10.	Methyl jasmonate (most of the plants)	Detaches HKII from VDAC and causes loss of mitochondrial function;	1	In vitro and in vivo study in mice model	[172,173]
11.	Withanone (*Withania somnifera*)	Acts as a ROS-producing agent causing DNA and mitochondrial damage	3	In vitro study	[174]
12.	Xanthones *(Garcinia mangostana*)	Causes loss of mitochondria membrane potential	6	In vitro and in vivo study	[175,176]

**Table 3:** Categorization of plant extracts into different classes of mitocans.

S. No.	Anticancer Plant	Extract	Cancer Type/ Cell Lines	Mode of Action	Proposed Class	References
1.	American Ginseng (*Panax quinquefolis*)	Steamed and extracted by ethanol	Colon cancer (SW-480)	Decreases the expression of Bcl-2 and induce mitochondrial-mediated apoptosis	2	[177]
2.	Ashwagandha (*Withania somnifera*)	Methanolic	Breast cancer (MCF-7)	Acts as a ROS-producing agent causing DNA and mitochondrial damage	3	[174]
3.	Barbed skullcap (*Scutellaria barbate*)	Aqueous	Primary liver cancer mouse hepatoma cells (H22)	Apoptosis via loss of mitochondrial transmembrane potential, the release of cytochrome c, and activation of caspase-3	6	[178]
4.	Bitter gourd *(Momordica charantia)*	Methanolic	Human nasopharyngeal carcinoma cells (Hone-1), gastric adenocarcinoma cells (AGS), colon cancer cells (HCT-116), and lung adenocarcinoma cell (CL1-0)	Increased Bax/Bcl-2 ratio and mitochondria-dependent apoptosis	2	[179]
5.	Cape gooseberry (*Physalis peruviana*)	Ethanol	Human hepatocellular carcinoma (Hep G2 cells) + mouse model	Apoptosis mediated through a mitochondrial signaling transduction pathway	2	[180]
6.	Cinnamon (*Cinnamomum cassia*)	Aqueous	Cervical cancer (SiHa)	Induces apoptosis by loss of mitochondrial membrane potential (MMP)	6	[181]
7.	Fish mint (*Houttuynia cordata*)	Ethanol	Human colon adenocarcinoma (HT-29 cells)	Loss of mitochondria membrane potential increased ROS production and alterations ofmitochondrial proteins such as cytochrome c, Apaf-1, AIFand pro-caspase-9	6	[182]
8.	Ginger (*Zingiber officinale*)	Methanol	Prostate cancer	Altered Bax/Bcl-2ratio and collapse of mitochondrial membrane potential	2	[183]
9.	Gotu Kola (*Centella Asiatica*)	Methanol	Breast cancer (MCF-7)	Loss of mitochondria membrane potential due to increased expression of Bax and decreased expression of Bcl-2	2	[184]
10.	Huaier (*Trametes robiniophila murr*)	Aqueous	Breast cancer (MCF-7 and MDA-MB-231)	Suppresses the Bcl-2 expression and up-regulate Bax expression and leads to mitochondrial-mediated apoptosis	2	[185]
11.	Ironwood (*Memecylon edule*)	Ethyl acetate	Human gastric carcinoma	Apoptosis by decreasing the expression of anti-apoptotic protein Bcl-2	2	[186]
12.	Pupa grass (*Cordyceps militaris*)	Aqueous	Breast cancer (MDA-MB-231 cells)	Activation of caspase-3 and mitochondria dysfunctions	2	[187]
13.	Slender red seaweed (*Gracilaria tenuistipitata*)	Ethanol	Oral squamous cell cancer (Ca9-22 cell)	Inhibition and apoptosis by increased ROS level, GSH depletion, caspase activation, and mitochondrial depolarization	6	[188]
14.	Veldt grape (*Cissus quadrangularis Linn*)	Acetone	Skin cancer (A431)	Altered Bax/Bcl-2ratio, release of cytochrome c from mitochondria	2	[189]
